# Does organizational context influence the use of best practices by healthcare aides in residential long term care?

**DOI:** 10.1186/1748-5908-10-S1-A64

**Published:** 2015-08-20

**Authors:** Carole A Estabrooks, Janet E Squires, Leslie Hayduk, Debra Morgan, Greta G Cummings, Liane Ginsburg, Peter G Norton

**Affiliations:** 1 Faculty of Nursing, University of Alberta, Edmonton, Alberta, T6G 1C9, Canada; 2 School of Nursing, Faculty of Nursing, University of Ottawa, Ottawa, Ontario, K1N 6N5, Canada; 3 Ottawa Hospital Research Institute, Ottawa, Ontario, K1Y 4E9, Canada; 4 Department of Sociology, University of Alberta, Edmonton, Alberta, T6G 2H4, Canada; 5 Canadian Centre for Health and Safety in Agriculture, University of Saskatchewan, Saskatoon, Saskatchewan, S7N 2Z4, Canada; 6 School of Health Policy & Management, York University, Toronto, Ontario, M3J 1P3, Canada; 7 Department of Family Medicine, University of Calgary, Calgary, Alberta, T2N 1N4, Canada

## Introduction

Knowledge translation researchers in the last decade have seen a torrent of publications arguing for the central importance of organizational context in influencing implementation success. The Translating Research in Elder Care (TREC) program investigates the role of organizational context on the use of best practices, provider and resident outcomes in residential long-term care facilities (nursing homes) in three western Canadian provinces. The purpose of this presentation is to present findings from our study examining the influence of organizational context on the use of best practices by healthcare aides.

## Methods

We collected survey data from 36 nursing homes, 103 units and 1262 healthcare aides and used Hierarchical Linear Modeling (HLM) to assess elements of organizational context that predicted best practice use by healthcare aides. Organizational context was measured using the Alberta Context Tool (ACT).

## Findings

In the final models, instrumental use of best practices was predicted by age, sex, shift worked, job efficacy, belief suspension, and the contextual variables social capital, organizational slack, informal interaction frequency, unit type, and profit status; 18% of the variance was explained in the final model. Conceptual use of best practices was predicted by: English language status, job efficacy, belief suspension, intent to use research, number of information sources uses, perception of adequate knowledge, and the contextual elements leadership, feedback processes, organizational slack, availability of resources, and province; 43% of the variance was explained in the final model.

## Conclusions

Our study identifies several individual and contextual factors that play an important role in healthcare aides' use of best practices in nursing homes. This has important practical implications for designing strategies for dissemination and implementation of best practices to increase healthcare professionals' ability to practice evidence informed care to improve health outcomes.

Funded by Canadian Institutes of Health Research (CIHR).

